# Primary ciliary dyskinesia

**DOI:** 10.1093/pch/pxae102

**Published:** 2025-04-03

**Authors:** Vincent Lavoie, Zofia Zysman-Colman, Adam J Shapiro

**Affiliations:** McGill University Health Centre, Montreal, Quebec; McGill University Health Centre, Montreal, Quebec; McGill University Health Centre, Montreal, Quebec; Research Institute of the McGill University Health Centre, Montreal, Quebec

**Keywords:** *Bronchiectasis*, *Primary ciliary dyskinesia*, *Situs inversus totalis*

**What’s New**

- Primary ciliary dyskinesia (PCD) remains underdiagnosed, although its prevalence is greatly increasing, particularly in Canadian Indigenous populations.
- Key clinical features (neonatal respiratory distress, early-onset, year-round wet cough or sino-nasal congestion, or organ laterality defect(s)) should prompt referral for PCD diagnostic testing.
- Current PCD diagnostic testing in Canada includes nasal nitric oxide measurement with confirmation by genetic testing and/or ciliary transmission electron microscopy.
- Therapeutic goals in PCD include slowing disease progression, treating acute respiratory exacerbations, preventing complications, and improving quality of life until effective, personalized therapies become available.

## BACKGROUND

Primary ciliary dyskinesia (PCD) is an inherited disease of motile cilia, characterized by neonatal respiratory distress, chronic sino-oto-pulmonary infections, left-right organ laterality defects, and subfertility (1). The classic Kartagener Syndrome triad of *situs inversus totalis*, bronchiectasis, and chronic sinusitis frequently occurs in PCD yet is not always present, especially in children. This review highlights the multitude of symptoms that should prompt referral for PCD in pediatric practice, diagnostic tests for PCD confirmation, standard PCD therapies that pediatricians may encounter, and research into personalized treatments for patients with PCD.

## PCD PREVALENCE

With genetic discovery over the past decade, the estimated prevalence of PCD has risen from 1:15,000-1,30,000 to 1:7600 people (2,3). Through founder variants or consanguinity, this prevalence may be even higher in specific populations, including Canadian Inuit and South Asian communities. In these groups, PCD prevalence may approach 1:1400 to 1:2200 patients (4,5), respectively, which is similar to the cystic fibrosis (CF) prevalence in White Canadians (6). In fact, the prevalence of PCD in Canadian Inuit represents the highest prevalence of any group worldwide and likely extends across the Arctic (7). PCD also occurs in Canadian First Nations through a likely genetic founder variant from Cree origins (8). Overall, PCD remains underdiagnosed with approximately 50,000 patients estimated across North America but less than 2000 definitively diagnosed per PCD Foundation estimates. Many of these people are misdiagnosed or followed as cases of idiopathic bronchiectasis, without receiving appropriate PCD care (9).

## PATHOPHYSIOLOGY

**Figure 1.**
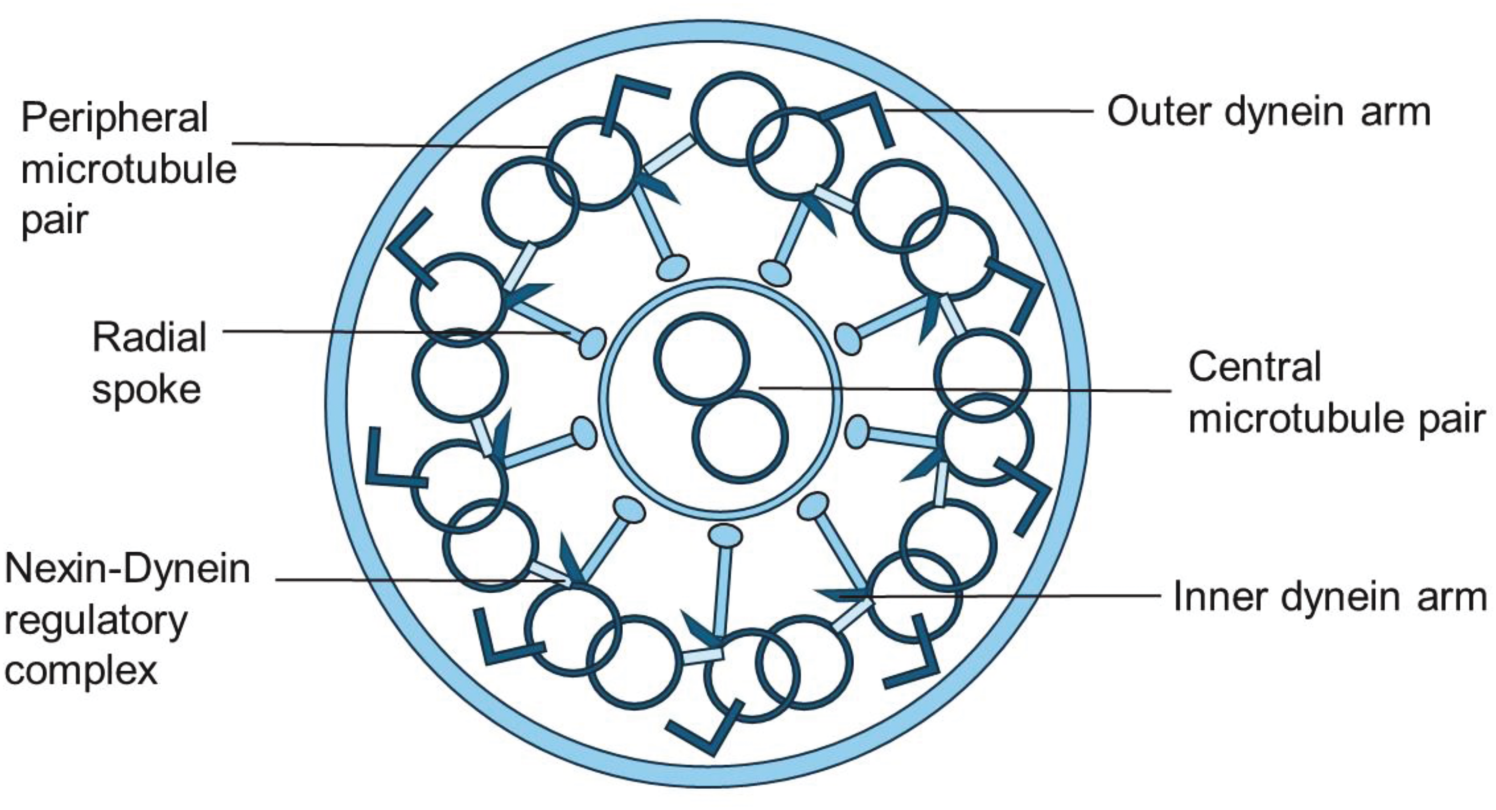
Ultrastructure of a normal ciliary axoneme. Cross-section of a normal ciliary axoneme with 9 + 2 configurations, showing the various ultrastructural components that power ciliary beat (outer and inner dynein arms), drive normal axonemal formation along its length (nexin-dynein regulatory links), and provide best stability (radial spokes and central apparatus).

Motile cilia are hair-like structures at the apical surface of cells lining the upper and lower respiratory tract. Approximately 200 cilia per epithelial cell sweep fluid, mucus, and inhaled particulates/organisms along the airway surface to be expectorated or swallowed. Motile cilia are also crucial in cerebrospinal fluid movement and gamete propulsion in males and females. Each cilia is composed of nine peripheral microtubule doublets encircling a central pair of microtubules (classic 9 + 2 configuration), outer and inner dynein arms, nexin-dynein regulatory complexes, and radial spokes (10,11) (Figure 1). Dyskinetic ciliary beating due to a genetic variant affecting proteins in any of these structures, or decreased ciliary numbers from variants in oligociliary genes, cause ineffective mucociliary clearance, leading to chronic inflammation, bacterial superinfection, irreversible airway dilatation/damage (bronchiectasis), and sometimes respiratory failure (12).

## CLINICAL FEATURES

The classic respiratory symptoms in PCD start in infancy, often immediately at birth, and typically do not wait to present in later childhood. The North American Genetic Diseases of Mucociliary Clearance Consortium (GDMCC) recognizes four key clinical symptoms that are highly sensitive and specific for PCD in children, including (1) neonatal respiratory distress requiring oxygen therapy or non-invasive ventilation for ≥24 h in a full-term newborn, (2) year-round wet cough starting within the first 6 months of life, (3) year-round sino-nasal congestion starting within the first 6 months of life, or (4) any organ laterality defect (*situs inversus totalis* or *situs ambiguus*) (13). The presence of any two of these key clinical symptoms confers a sensitivity of 80% for having PCD, while the presence of all four key symptoms is 99% specific for the disease (13). The PICADAR clinical scoring instrument is also highly predictive of PCD in children but relies heavily upon organ laterality defects and may be less accurate in cases of PCD with normal organ arrangement (14). Specific combinations of symptoms, including *situs inversus totalis* with neonatal respiratory distress in a term infant, strongly suggest PCD (15) (Table 1). In children with unexplained, chronic respiratory issues but no apparent laterality defects on chest radiography, abdominal and cardiac imaging demonstrating an organ laterality defect can greatly increase the suspicion of PCD (17).

**Table 1.** Key clinical symptoms of primary ciliary dyskinesia (PCD) in newborns, children, and adults*

When to suspect primary ciliary dyskinesia in newborn, pediatric, and adolescent/young adult patients
*Symptoms highly suggestive of PCD in newborns* ^†^: ○ *Situs inversus totalis***and** neonatal respiratory distress^‡^ ○ *Situs ambiguus***and** neonatal respiratory distress^‡^ without cardiac defect ○ *Situs ambiguus***and** neonatal respiratory distress^‡^ out of proportion to cardiac defect ○ Neonatal respiratory distress^‡^ at term birth, requiring supplemental oxygen or positive pressure support for ≥24 h **and** lobar atelectasis on chest radiography ○ Neonatal respiratory distress^‡^ at term birth, requiring supplemental oxygen or positive pressure support for ≥24 h **and** family history of PCD, chronic sino-oto-pulmonary disease, or unexplained bronchiectasis
*Symptoms highly suggestive of PCD in children*: ○ At least 2 of 4 key clinical symptoms: ▪ Year-round wet cough with onset before 6 months of age or ▪ Year-round nasal congestion with onset before 6 months of age or ▪ Neonatal respiratory distress^‡^ at term birth, requiring supplemental oxygen or positive pressure support for ≥24 h or ▪ An organ laterality defect ○ Unexplained bronchiectasis** with chronic sino-oto-pulmonary disease since early childhood or family history of PCD
*Symptoms highly suggestive of PCD in adolescents/young adults*: ○ Unexplained bronchiectasis** and at least 1 of 6 key clinical symptoms: ▪ Chronic rhino-sinusitis or ▪ Ongoing otitis/effusion in adolescence/adulthood or ▪ An organ laterality defect or ▪ Male or female infertility ▪ Chronic sino-oto-pulmonary symptoms since early childhood or ▪ Family history of PCD

*Version adapted from Wee et al., *Primary Ciliary Dyskinesia*, Pediatrics (2024) (15);

^†^Prenatal cerebral ventriculomegaly, presumably from ependymal ciliary dysfunction in brain ventricles, has been described in newborns with PCD. However, this finding has not been verified as predictive of PCD diagnosis versus controls (16);

^‡^In PCD, the neonatal respiratory distress often occurs 12 to 24 h after birth and is accompanied by shifting, lobar atelectasis on chest radiography;

**Bronchiectasis in PCD is more prominent in the middle/lower lobes.

Neonatal respiratory distress is present in up to 80% of neonates with PCD (18). Compared to other causes of neonatal respiratory distress, the onset of PCD tends to be delayed, often presenting at 12 to 24 h of life or later. Up to 70% of newborns also show lobar atelectasis of the upper or middle lobes on chest radiography, which often appears days after the onset of distress and may be prolonged, especially in premature neonates with PCD (16,18). On average, term babies with PCD and neonatal respiratory distress require 2 weeks of respiratory support, and home supplemental oxygen may be needed (18). Prenatal cerebral ventriculomegaly is reported in limited cases of PCD (16), and hydrocephalus may rarely arise in some cases with variants in specific genes (19–21).

At birth or soon thereafter, year-round nasal congestion appears with eventual chronic sinusitis. This issue very often occurs on a daily basis and never fully resolves, even after antibiotic therapy (22). Nasal polyps may occur with age but are less common compared to patients with CF (23). Year-round wet cough, similarly occurring on a daily basis without complete resolution, also appears in early infancy. Chronic sputum production is common. Recurrent lower respiratory tract infections are also common, leading to decreased pulmonary function and the development of bronchiectasis that typically affects the lower and middle lobes. Poor clinical outcomes, including more advanced bronchiectasis, faster decline in pulmonary function, and poor physical growth are associated with specific PCD genotypes (*CCDC39*, *CCDC40*) (24,25). Conversely, patients with variants in *DNAH11* show relative preservation of lung function and a lower prevalence of neonatal respiratory distress (26).

Outside of the respiratory tract, ciliary dysfunction in eustachian tubes leads to recurrent otitis media and/or persistent middle ear effusions in the vast majority of children with PCD (27). These issues commonly result in conductive hearing loss and/or speech delay (28,29). Repeat placement of middle ear ventilation tubes in childhood and even into adulthood is often required to treat effusions.

Organ laterality defects, caused by dysfunction of nodal cilia in developing embryos, occur in approximately 50% of PCD cases. Laterality defects may range from classic *situs inversus totalis* (mirror image reversal of all organs) to *situs ambiguus* (left-right patterning defects falling on a spectrum between normal and complete reversal) in 12% to 20% of patients (17,30). Patients with *situs ambiguus* may display collections of organ defects (e.g., left or right isomerism), single organ defects (e.g., isolated dextrocardia or isolated asplenia/polysplenia), or complex congenital cardiovascular defects (heterotaxy) (31) (Table 2). Children with complex cardiovascular or splenic defects seem to have worse long-term respiratory and nutritional outcomes (32).

**Table 2.** Possible organ laterality defects with situs ambiguus in PCD

*Cardiac defects* Isolated dextrocardia Simple congenital heart defects (ASD, VSD, etc.) Complex congenital heart defects (heterotaxy) Atrial isomerism Common atrium Atrioventricular discordance Ventriculoarterial discordance	*Abdominal defects* Situs inversus abdominalis Midline liver Dextrogastria Polysplenia or asplenia (right- or left-sided) Intestinal malrotation Horseshoe kidney Annular pancreas Duodenal atresia Fused adrenal glands Extrahepatic biliary atresia
*Vascular defects* Right aortic arch Bilateral or left superior vena cava Interrupted inferior vena cava Levotransposition or dextrotransposition of the great vessels Anomalous pulmonary venous return	*Pulmonary defects* Left pulmonary isomerism Right pulmonary isomerism Pulmonary situs inversus

*ASD atrial septal defect*; *VSD ventricular septal defect*.

Rare syndromic forms of PCD also exist, including X-linked retinitis pigmentosa due to variants in *RPGR* (33,34), X-linked oro-facial-digital syndrome through *OFD1* variants, autosomal recessive lissencephaly with the *TP73* gene, and Cri-du-Chat syndrome through hemizygous inheritance of a single variant in *DNAH5* plus the characteristic 5p deletion, which includes the same allele, on the opposite chromosome (35).

## DIAGNOSIS

Diagnosis of PCD was difficult before genetic discovery defined many of the underlying genes. As there is no single test that can detect all cases of PCD, patients often require several tests for a diagnosis, including genetic testing, ciliary ultrastructural analysis on transmission electron microscopy (TEM), and nasal nitric oxide (nNO) measurement. None of these tests can rule out PCD. Additional testing limited to research laboratories includes high-speed videomicroscopy analysis of ciliary beat pattern and ciliary protein immunofluorescence.

**Figure 2.**
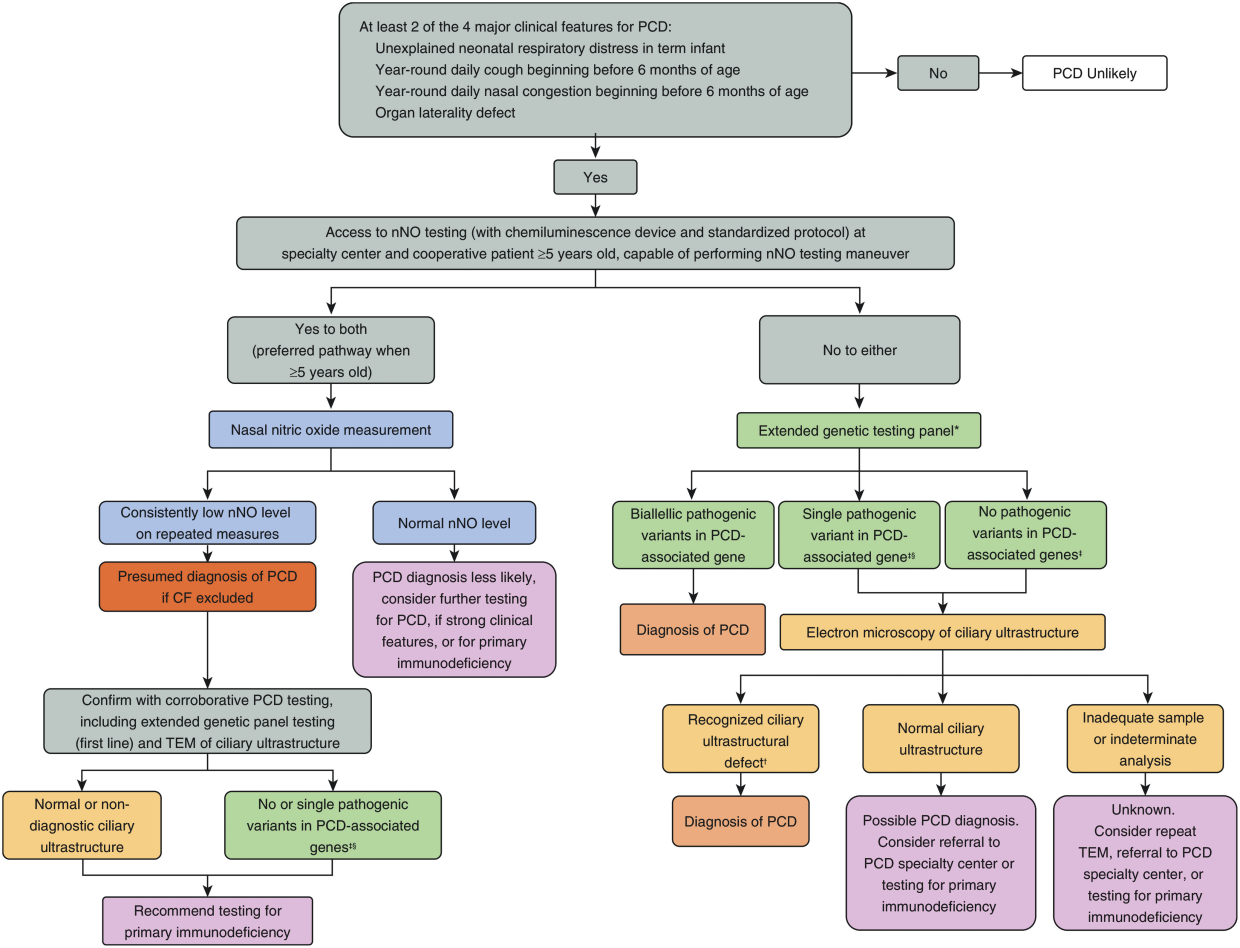
American Thoracic Society suggested algorithm for evaluating patients with suspected primary ciliary dyskinesia. Reprinted with permission of the American Thoracic Society. Copyright © 2024 American Thoracic Society. All rights reserved (36,37). The American Journal of Respiratory and Critical Care Medicine is an official journal of the American Thoracic Society.

Clinical practice guidelines suggest referral for possible PCD in patients with an appropriate PCD clinical phenotype, including at least two key clinical PCD symptoms (36) (Figure 2). In patients ≥5 years old who can cooperate, nNO measurement by exhalation against resistance at a PCD Foundation accredited centre is the preferred initial PCD investigation in North America. For unexplained reasons, nNO values measured by chemiluminescence devices, with approved protocols, are greatly reduced in patients with PCD. Repeatedly low nNO values (<77 nL/min) are highly sensitive and specific for PCD (38,39). Up to 10% of patients with PCD may have nNO values above the 77 nL/min cutoff, so normal nNO levels do not rule out PCD (40). Some patients with CF, rare combined immunodeficiencies, or acute viral respiratory infections may also have reduced nNO values (40–43). Thus, CF testing should be negative when using nNO testing to investigate PCD, and patients must be free from viral infection for at least 2 weeks before testing. Children <5 years old can undergo nNO testing using a tidal breathing technique, but cutoff values are not robustly validated with this technique (39).

When nNO values are repeatedly low, or if nNO testing is unavailable, genetic testing is preferred for diagnostic confirmation. Pathogenic variants in >50 unique genes cause PCD (44) (Table 3). Most commercial genetic panels analyze >40 PCD-causing genes, and genetic testing confirms 70% to 80% of PCD cases (46,47). Diagnostic accuracy may improve with exome or genome sequencing (48). Most PCD-causing genes are inherited in an autosomal recessive manner, with a few X-linked genes and a single autosomal dominant gene that arises de novo. Thus, biallelic, pathogenic variants in a single PCD gene, or a single pathogenic variant in an X-linked or autosomal dominant gene, are definitively diagnostic of PCD.

**Table 3.** Ultrastructure defects and PCD genotypes with their expected nasal nitric oxide levels and association with organ laterality defects*

Ultrastructure defects	Gene	nNO	Laterality defect
Outer dynein arm defect 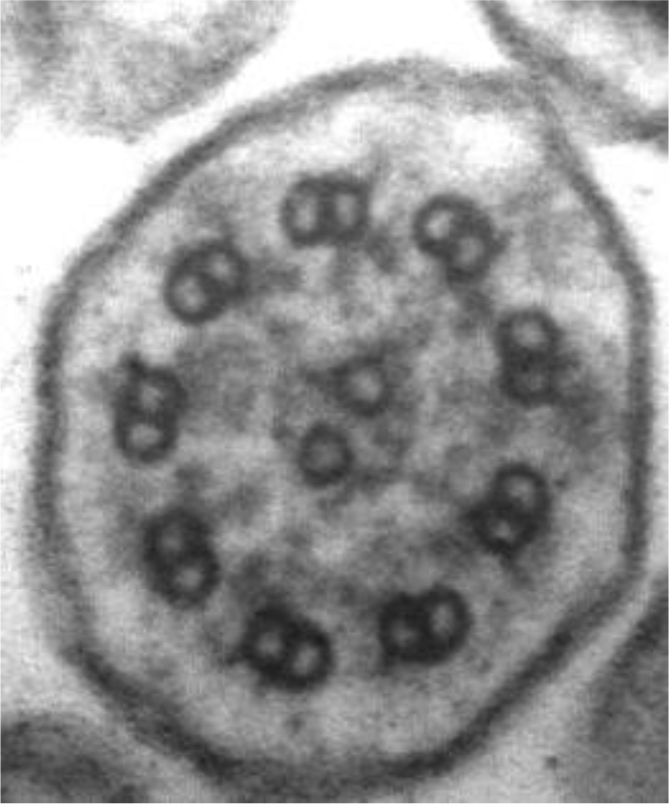	*ARMC4* *CCDC103* *CCDC114* *CCDC151* *CLXN* *DNAH1* *DNAH5* *DNAH9* *DNAI1* *DNAI2*	Low Low/normal Low NR NR NR Low Low/normal Low NR	Yes Yes Yes Yes Yes Yes Yes Yes Yes Yes
Outer and inner dynein arm defect 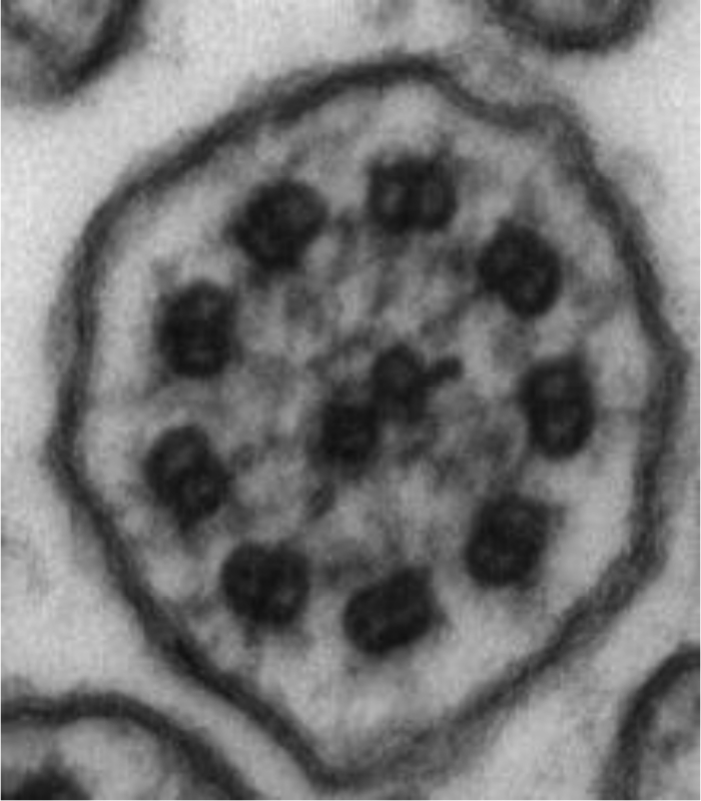	*CFAP298* *CFAP300* *DNAAFI1* *DNAAF2* *DNAAF3* *DYX1C1* *HEATR2* *LRRC6* *PIH1D3* *SPAG1* *ZYMND10*	Low Low NR Low Low Low Low Low Low Low Low	Yes Yes Yes Yes Yes Yes Yes Yes Yes Yes Yes
Inner dynein arm defect with microtubule disorganization 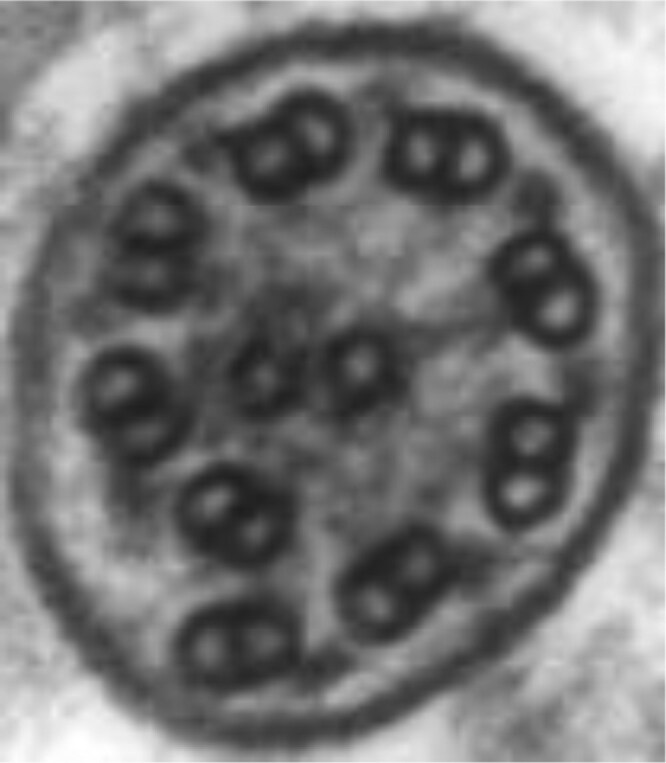	*CCDC39* *CCDC40* *TTC12*	Low Low Low/normal	Yes Yes NR
Normal/near normal 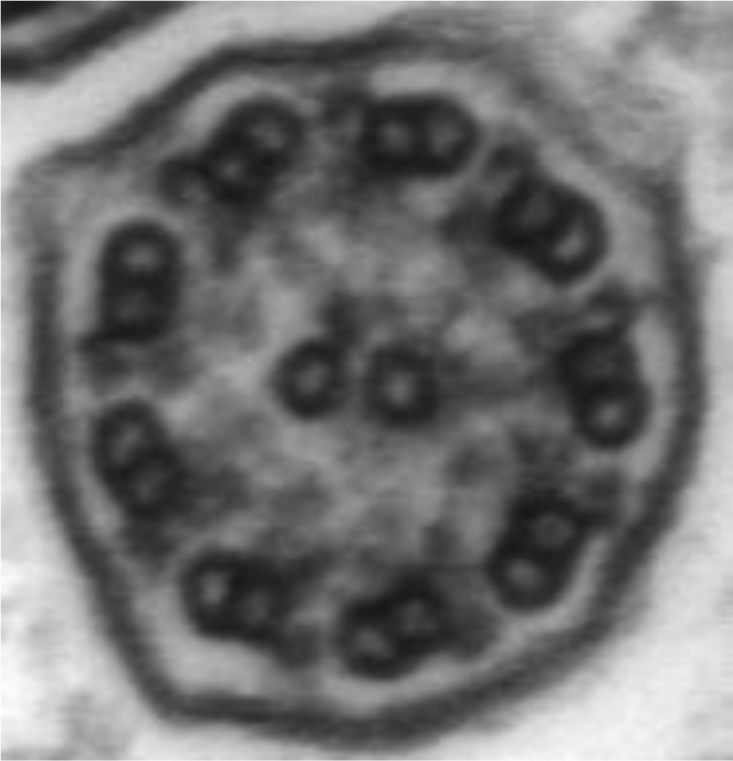	*CCDC164* *CCDC65* *CFAP53* *CFAP57* *CFAP74* *DNAH11* *DNAJB13* *GAS2L2* *LRRC56* *NEK10* *NME5* *OFD1* *RPGR* *TP73* CCNO^†^ *FOXJ1*^†^ MCIDAS^†^ *CFAP221*^‡^ DNAJB13^‡^ HYDIN^‡^ *RSPH1*^‡^ RSPH3^‡^ RSPH4A^‡^ RSPH9^‡^ SPEF2^‡^ STK36^‡^	Low Low Normal Low Normal Low Low Low/normal Normal Normal NR Low Normal NR Low Normal Low Normal Low Low/normal Low/normal Low Low Low/normal Low Normal	NR NR Yes NR NR Yes NR NR Yes NR NR Yes NR NR NR Yes NR NR NR NR NR NR NR NR NR NR

*Version adapted from Wee et al., *Primary Ciliary Dyskinesia*, Pediatrics (2024) (15);

^†^CCNO, FOXJ1, MCIDAS genes result in oligociliary defects with only one to a few cilia per epithelial cell, which appear to have normal ultrastructure on TEM if located and examined;

^‡^These genes are associated with central apparatus and radial spoke defects. However, these defects cannot be reliably diagnosed on TEM as the majority of cross-sections often appear normal or non-diagnostic (45).

If genetic testing is inconclusive, ciliary analysis with transmission electron microscopy (TEM) should be performed on respiratory epithelial cells looking for disease-causing ultrastructural defects. However, TEM analysis is difficult to reliably perform outside of a few, expert Canadian centres and may be normal or non-diagnostic in 30% of PCD cases (45,49). In 20% to 30% of PCD patients, both TEM and genetic testing may be inconclusive, with repeatedly low nNO values as the only positive PCD test. In these cases, empiric therapy for PCD is warranted but providers must evaluate for other conditions, including CF and immunodeficiency.

### Therapy and monitoring

There is no therapy to correct the underlying ciliary dysfunction in PCD. Thus, treatment strategies include slowing disease progression, treating acute respiratory exacerbations, preventing complications, and improving quality of life. Many PCD therapies are borrowed from the management of CF and modified per PCD Foundation consensus, which is based on a combination of evidence and expert opinion (50).

Regular airway clearance is the foundation of PCD therapy, including manual chest percussion, handheld expiratory resistance devices (i.e., Positive Expiratory pressure Therapy - PEP therapy), oscillatory vests, or intense cardiovascular exercise once to twice daily (51). Many PCD providers prescribe daily, nebulized hypertonic saline (3% to 7%) to promote coughing with secretion mobilization. Though inhaled hypertonic saline does not improve pulmonary function in clinical trials (52), it does have a limited impact on respiratory-related quality of life (53). Other inhaled mucolytics, including dornase alpha, remain unstudied in PCD but worsen respiratory outcomes in patients with non-CF bronchiectasis (54).

Patients with PCD expect a daily, wet cough at baseline. When this cough increases for several days, or when other respiratory symptoms present (e.g., change in sputum quality or amount, chest pain, dyspnea, etc.), patients need treatment for a respiratory exacerbation. Acute therapy with oral antibiotics targeting microbes in recent sputum cultures plus the increased frequency of regular airway clearance is appropriate for mild exacerbations. More severe exacerbations, including worsening dyspnea, supplemental oxygen requirement, or a large decrease in pulmonary function, require hospitalization and intravenous antibiotics with escalation of daily airway clearance. Typically, antibiotic therapy is prolonged to 2 to 3 weeks duration in PCD (50), though the majority of patients receiving intravenous antibiotic therapy regain 90% of their respiratory function after 1 week of treatment (55).

Chronic macrolide anti-inflammatory therapy is beneficial in PCD, and thrice weekly azithromycin decreases acute respiratory exacerbations by 50% over a 6-month period (56). Though there is a risk of antibiotic resistance with azithromycin therapy, this did not occur during 6 months of observation. Azithromycin is often used in patients with PCD and an elevated burden of respiratory exacerbations (≥2 to 3 outpatient exacerbations or ≥1 inpatient exacerbation per year).

Otolaryngology therapies are essential for children with PCD, due to the abundant presence of chronic rhino-sinusitis and otitis media with effusion. Children should be followed by an otolaryngologist at least one to two times per year for conductive hearing loss and/or persistent ear effusions. As some children with PCD are unaware of their hearing deficits (57), audiology is recommended in all patients at diagnosis, with repeat assessments thereafter per otolaryngologists. Pressure equalization tubes (PET) are advocated for children with PCD and conductive hearing loss or speech delay from middle ear effusions (50,57). Though PET placement PCD risks recurrent otorrhea, this is usually controllable with short-term, topical drops (57). Children with PCD should also be monitored for nasal disease and perform nasal hygiene with rinses, antibiotics, or nasal steroids, as directed. In adolescence, functional endoscopic sinus surgery may be appropriate for poor quality of life from chronic sinusitis (29,57).

Long-term follow-up of PCD is crucial to prevent pulmonary damage and functional deterioration. Experts recommend follow-up with a multidisciplinary team (respirologists, otolaryngologists, physiotherapists, respiratory therapists, nurses, and social workers) in a PCD Foundation clinical centre or a CF centre (50). Patients should be seen at least two to four times per year with spirometry and sputum cultures at each visit. While *Pseudomonas aeruginosa* is not specifically proven to worsen lung function in PCD, eradication therapy should be strongly considered when found in sputum cultures (58). Other gram-negative opportunistic organisms, including *Burkholderia cepacia* complex, have been reported in PCD sputum, may cause worsened respiratory outcomes, and should be considered for eradication (59). Chest radiography should be performed at diagnosis, during acute respiratory exacerbations, and every 2 to 4 years in stable patients. To quantify and follow bronchiectasis, chest computed tomography is considered at least once when patients are old enough to cooperate and avoid anesthesia. As congenital heart defects are 200 times more common in PCD, and anatomic spleen anomalies may result in functional asplenia, both echocardiograms and abdominal imaging should be considered in all patients with PCD, with functional spleen testing when anomalies are found (17,60).

Standard vaccinations per local schedules are recommended in PCD. In addition, patients should receive annual influenza vaccination, SARS-CoV-2 vaccines, additional pneumococcal vaccines, and RSV prophylaxis per eligibility (61–63).

### Future of PCD

Innovative and personalized PCD therapies are currently being developed, including inhaled mRNA therapeutics to correct the underlying gene-specific protein defect in ciliary ultrastructural components. Pre-clinical studies are ongoing with an inhaled liposomal nanoparticle-mRNA complex for the correction of pathogenic *CCDC40* variants (64). A phase 1, randomized, double-blind, placebo-controlled study is also ongoing with inhaled mRNA therapy for the correction of *DNAI1* variants (65). Several international research groups support PCD efforts worldwide, including the PCD Foundation and the Genetic Disorders of Mucociliary Clearance Consortium in North America, and the International PCD cohort, European Reference Network-LUNG PCD Core, and Better Experimental Approaches to Treat PCD (BEAT-PCD) networks in Europe. These groups are working to populate clinical registries that will be essential for future clinical trials and continued patient referrals for PCD diagnosis are critical to fill these.

## CONCLUSION

PCD is an increasingly well-recognized pediatric disease, particularly in Canadian indigenous populations, that is underdiagnosed due to challenges in recognizing its phenotype and accessing effective diagnostic testing. Clinicians should consider this disease and pursue appropriate testing when key clinical symptoms are present at different stages of pediatric development. Consultations with pediatric respirologists, geneticists, or PCD Foundation-accredited clinics are critical for accurate diagnosis and follow-up of children with PCD. Genotyping patients with PCD is essential to fulfill enrollment in trials investigating personalized, gene-specific therapies, which have the potential to drastically improve the lives of children with PCD.
